# Kidney clearances of protein-bound uremic toxins predict outcomes in chronic kidney disease: a prospective cohort study

**DOI:** 10.1080/0886022X.2025.2578418

**Published:** 2025-11-03

**Authors:** Danshu Xie, Mengdi Jiang, Jiaolun Li, Yingjie Chen, Feng Ding, Wenji Wang

**Affiliations:** Department of Nephrology, Shanghai Ninth People’s Hospital, Shanghai Jiao Tong University School of Medicine, Shanghai, P.R. China

**Keywords:** Protein-bound uremic toxins, kidney clearance, chronic kidney disease, outcomes

## Abstract

This prospective cohort study investigated the kidney clearance of protein-bound uremic toxins (PBUTs)—specifically, indole sulfate (IS), p-cresol sulfate (pCS), and indole-3-acetic acid (IAA)—across various stages of chronic kidney disease (CKD) stages and their associations with adverse clinical outcomes. From July 2018 to December 2020, 186 non-dialysis CKD patients were enrolled and followed until June 2025. Serum and 24-hour (24 h) urine PBUT levels were measured, and kidney clearances were analyzed. The findings indicated a decrease in the 24 h kidney clearances of IS (Cis), pCS (Cpcs), and IAA (Ciaa) corresponding with a reduction in glomerular filtration rate (GFR) across the CKD stages. For instance, the clearance of indoxyl sulfate was observed to decline from 26.7 mL/min in stage 1 to 2.2 mL/min in stage 5. PBUT clearances and fractional clearances were generally unaffected by 24 h urinary protein levels, with the exception of fractional clearance of IAA. Elevated levels of Cis and Ciaa were independently correlated with decreased risks of renal outcomes (IS: HR 0.964; IAA: HR 0.941) and hospitalization (IS: HR 0.984; IAA: HR 0.961). The study concluded that the 24 h kidney clearances of IS and IAA are independently associated with renal adverse outcomes and hospitalization, with minimal influence from urinary protein excretion.

## GRAPHIC_ABSTRACT

**Figure:**
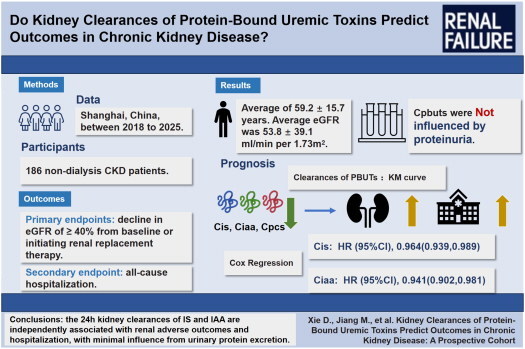

## Introduction

The prevalence of CKD is more than 10% worldwide and 8.2–10.8% in China [1–3]. Chronic kidney disease (CKD) is characterized by the accumulation of metabolic solutes (such as uremic toxins), alongside exogenous drugs or toxins, resulting from compromised glomerular filtration and tubular secretion functions [4–6]. Approximately 25% of the currently identified uremic toxins are protein-bound uremic toxins (PBUTs), which exhibit a protein-bound fraction exceeding 90% and are predominantly secreted through renal tubules [7,8]. In the past decade, PBUTs, including indole sulfate (IS), *p*-cresol sulfate (pCS), and 3-indole acetic acid (IAA), have gained significant attention [9,10] because of their significantly elevated concentrations in serum in CKD and their toxic effects on various physical systems [8–10]. Furthermore, elevated serum concentrations of PBUTs have been correlated with the progression and unfavorable prognosis of CKD [9,11,12], as well as an increased risk of cardiovascular events, as demonstrated by clinical trials [13,14].

In accordance with several established methods of estimated glomerular filtration rate (eGFR), as well as the urinary albumin to creatinine ratio (UACR) and markers of tubular injury, research has demonstrated their association with poor outcomes in CKD patients [15–17]. Nevertheless, the evaluation of tubular functions, particularly the secretion function, remains insufficient. The kidney clearance rates of PBUTs are determined by measuring their concentrations in both serum and urine, thereby facilitating the estimation of tubular secretion function [18,19].

To date, few studies systematically investigated the characteristics of PBUTs excreted by the kidneys at various stages of CKD. Furthermore, the relationship between the renal excretion of PBUTs and clinical outcomes in CKD remains inadequately understood. Recent research indicated the considerable interindividual variability in tubular secretion relative to glomerular filtration rate (GFR) among patients with chronic kidney disease (CKD), despite the moderate correlation between the clearance rates of protein-bound uremic toxins (PBUTs) and GFR [20,21]. The Chronic Renal Insufficiency Cohort (CRIC) study, which enrolled participants with an eGFR of between 20 and 70 mL/min per 1.73 m^2^, demonstrated that diminished renal clearance of IS was correlated with an elevated risk of CKD progression. Nevertheless, after adjusting for estimated glomerular filtration rate (eGFR), the analysis did not reveal a statistically significant association between the kidney clearance of endogenous secretory solutes, such as IS and pCS, and incidence of heart failure, myocardial infarction, or stroke [22,23].

Consequently, this study sought to investigate the characteristics of 24 h kidney clearance of IS, IAA, and pCS across different stages of CKD, and to assess their prognostic value in patients with non-dialyzed CKD.

## Methods

### Study population and definitions

**Figure 1.**
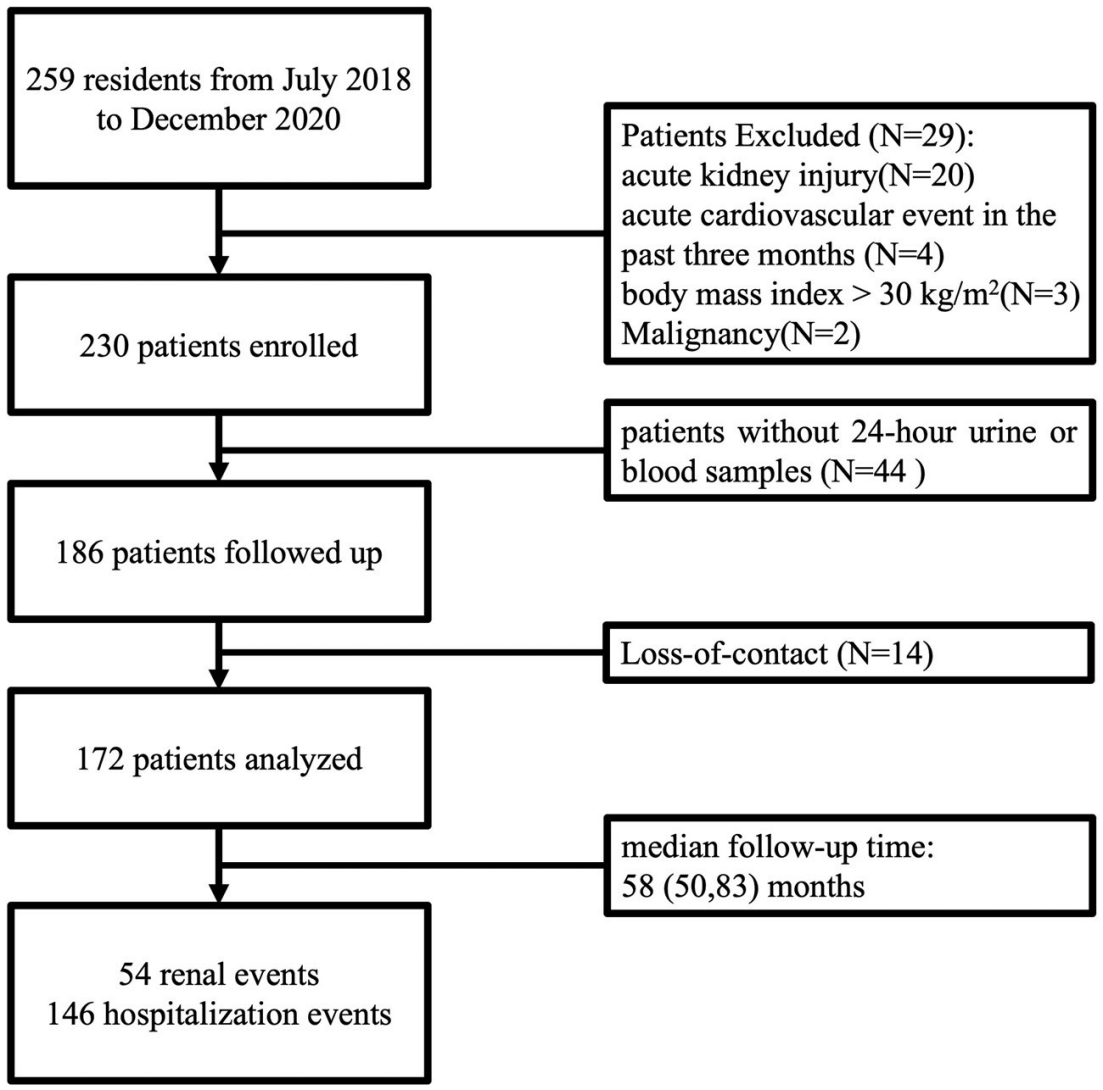
Flow chart of study progress. CKD: chronic kidney disease.

Between July 2018 and December 2020, a cohort of consecutive adult participants (aged ≥18 years) with non-dialyzed CKD (stage 1 to stage 5) was recruited. Exclusion criteria included patients with acute kidney injury, active infection (C-reactive protein >20 mg/L), inflammatory bowel disease or diarrhea, cirrhosis, liver dysfunction (alanine aminotransferase or aspartate aminotransferase ≥3 upper limit of normal or total bilirubin ≥34.2 μmol/L), acute cardiovascular events within the past three months, malignancy, body mass index >30 kg/m^2^, antibiotic or non-steroidal anti-inflammatory drugs within the past month, or pregnancy were excluded. CKD was diagnosed in accordance with the criteria established by the 2012 Kidney Disease: Improving Global Outcomes (KDIGO) [4]. The classification of CKD was determined based on an estimated glomerular filtration rate (eGFR), which was calculated utilizing the Chronic Kidney Disease Epidemiology Collaboration (CKD-EPI) equation [24]. A total of 259 patients were prospectively enrolled in the cohort. Among these, 29 patients were excluded from the study, and 44 patients did not submit the required 24 h urine or blood samples. Consequently, 186 patients with CKD were monitored until June 30, 2025. A total of 14 patients were lost to follow-up, resulting in 172 patients being available for prognostic analysis (Figure 1). The primary endpoint was defined as a composite of renal adverse outcome, characterized by sustained decline in eGFR of ≥ 40% from baseline or initiating renal replacement therapy (maintenance dialysis or kidney transplantation). The secondary endpoint assessed was all-cause hospitalization.

This study was conducted in compliance with the principles outlined in the Declaration of Helsinki and received approval from the Ethics Committee of Shanghai Ninth People’s Hospital, School of Medicine, Shanghai Jiaotong University (approval number: 2018-83-T74). Written informed consents were provided by all participants before their enrollment in the study, which was registered at www.clinicaltrials.gov (NCT 03711617). All protocols were executed in accordance with pertinent guidelines and regulations.

### Data and sample collection

The data collection process involved the extraction of information from electronic medical records by nephrologists, who subsequently entered the data into a computerized form using EpiData software. The recorded information encompassed demographic characteristics, comorbidities, medications, and renal function parameters, including serum creatinine, urea nitrogen, uric acid, and eGFR. Information pertaining to biochemical parameters, including serum alanine aminotransferase (ALT), albumin, high sensitive C-reactive protein (hsCRP), hemoglobin, and white blood cell counts, was also recorded.

Following patient enrollment, 24 h urine and serum samples were collected. The 24 h urine was considered incomplete as follows [25]: urine creatinine <5.3 mmol (600 mg)/24 h for female and <7.1 mmol (800 mg)/24 h for male. A portion of each biological sample was promptly sent to the hospital clinical laboratory, while the remaining aliquots of the sample were stored at −80 °C for subsequent analysis of PBUTs levels. The protein intake was estimated by calculating the ratio of urinary urea over urinary creatinine, a proxy for protein intake [26].

### Measurements of kidney clearances of PBUTs

IS, pCS, and IAA were selected, based on previously published evidence, including excretion mainly by known OAT1 and OAT3 on proximal tubules and minimal elimination by other organ systems. Serum and urine concentrations of IS, pCS, and IAA were determined using protein precipitation, after developing a targeted high performance liquid chromatography (HPLC) assay [27].

We focused on the kidney clearances of PBUTs, rather than serum concentrations, to avoid the potential impact of non-kidney factors on circulation levels. The 24 h kidney clearance rate of each PBUT (Cx) was calculated by the following formula:

$$C\left(x\right)=\frac{\text{Ux}(\mu \text{mol}/L)\times V(\text{mL})÷24÷60}{\text{Px}(\mu \text{mol}/L)}$$

Ux represented the concentration of x in the 24 h urine, V represented the 24 h urine volume, and Px represented the serum concentration of x. x referred to IS, pCS, or IAA.

Fractional clearance of each PBUT (FCx) was determined according to the formula:

$$\text{FC}\left(x\right)=\frac{\text{Ux}(\mu \text{mol}/L)÷\text{Px}(\mu \text{mol}/L)}{\text{Ucr}(\mu \text{mol}/L)÷\text{Pcr}(\mu \text{mol}/L)}$$

Ucr and Pcr represented the concentration of creatinine in serum and 24 h urine, respectively. Ux and Px represented the concentration of x in serum and 24 h urine, respectively. x referred to IS, pCS, or IAA.

### Statistical analyses

Continuous variables were expressed as means ± *SD*s or as medians (interquartile ranges), and categorical variables were expressed as frequencies (percentage). Comparisons of continuous and categorical data for patients in different groups were performed by the ANOVA test or the Kruskal-Wallis test and chi squared tests, respectively, as appropriate. The correlation among kidney clearances of PBUTs and eGFR was studied by the Spearman test.

Receiver-operating characteristics (ROC) curves and the areas under the curves (AUCs) were performed to evaluate the predictability of clearances of PBUTs, and the cutoff values were based on Youden index. Kaplan-Meier analyses were used to assess the differences in clinical prognosis between the groups with different clearances of PBUTs. Time to event analysis was performed for both serum PBUTs and kidney clearances of PBUTs using Cox proportional hazard models. Potential confounding variables were obtained by univariate Cox regression, and the multivariable Cox regression model consisted of variables with a *p*-value <0.2 in the univariate model. The robustness of the Cox regression outcomes was thoroughly evaluated *via* post-hoc power and sensitivity analyses. The power analysis was conducted using a two-tailed significance level of 0.05. The sensitivity analyses systematically investigated the proportional hazards (PH) assumption by employing Schoenfeld residuals. Moreover, we assessed the robustness of the primary association by incrementally incorporating variables into the adjustment model. This methodological approach enabled us to examine potential confounding factors and determine whether any variables functioned as intermediaries within the causal pathway.

All *p*-values were two tailed, and *p*-values <0.05 were considered significant. Statistical analyses were performed with SPSS v26.0 (IBM, New York) and R v4.5.1 (The R Foundation).

## Results

### Study population and characteristics

The study population had a mean age of 59.2 ± 15.7 years, with 69.4% (129 out of 186) being male. The average eGFR was 53.8 ± 39.1 mL/min per 1.73 m^2^ (Table 1). Age, serum urea, and creatinine levels exhibited a positive correlation with CKD stages, while hemoglobin levels showed a negative correlation. Significant variations were observed among groups with different CKD stages in terms of 24 h urinary protein excretion, as well as the prevalence of hypertension and diabetes. The ratio of urinary urea over creatinine concentration, an indicator of protein intake [28], was significantly lower in patients with CKD5 compared to those with CKD1 (27.6 ± 8.1 *vs.* 33.0 ± 11.8, *p* = 0.022) and CKD3 (27.6 ± 8.1 *vs.* 33.7 ± 12.2, *p* = 0.013), respectively. And with the progression of CKD, the utilization rate of renin-angiotensin system inhibitors (RASi) and oral hypoglycemic drugs (OHD) gradually declined, while the proportion of insulin use progressively increased.

**Table 1.** Clinical characteristics of study population.

	Total *n* = 186	CKD1 *n* = 45	CKD2 *n* = 33	CKD3 *n* = 36	CKD4 *n* = 34	CKD5 *n* = 38	*p-*Value
General							
Male, *n* (%)	129 (69.4)	29 (64.4)	21 (63.6)	28 (77.8)	24 (70.6)	27 (71.1)	0.682
Age, years	59.2 ± 15.7	49.5 ± 14.0	59.6 ± 15.5	60.8 ± 12.9	65.1 ± 13.8	63.4 ± 17.4	<0.001*
BMI, kg/m^2^	24.4 ± 3.7	23.9 ± 4.6	24.4 ± 3.1	25.4 ± 3.1	24.4 ± 3.8	23.7 ± 3.5	0.281
Smoking, *n* (%)	24 (12.9)	5 (11.1)	4 (12.1)	8 (22.2)	2 (5.9)	5 (13.2)	0.352
Diabetes, *n* (%)	70 (37.6)	7 (15.6)	8 (24.2)	19 (52.8)	15 (44.1)	21 (55.3)	<0.001*
Hypertension, *n* (%)	132 (71.0)	19 (42.2)	20 (60.6)	29 (80.6)	30 (88.2)	34 (89.5)	<0.001*
Laboratory parameters							
eGFR-EPI, mL/min/1.73 m^2^	53.8 ± 39.1	109.5 ± 11.9	74.4 ± 9.2	43.6 ± 8.5	21.2 ± 4.4	8.8 ± 3.2	<0.001*
Serum creatinine, µmol/L	138.0 (79.0, 301.5)	64.0 (52.0, 74.0)	89.0 (79.5, 101.5)	141.0 (121.5, 161.0)	243.0 (210.0, 307.5)	558.5 (414.3, 713.5)	<0.001*
Serum albumin, g/L	36 (31, 39.5)	37 (24, 41)	37 (28.5, 39.5)	35.7 ± 3.8	35.4 ± 4.5	34.1 ± 5.1	0.511
Hemoglobin, g/L	114.0 ± 26.9	129.5 ± 22.4	129.2 ± 21.7	118.1 ± 24.2	103.0 ± 18.9	88.6 ± 21.4	<0.001*
24-h urine total protein, mg	1394 (400, 6988)	900 (175, 2985)	1330 (175, 2285)	862 (260, 3110)	1385 (655, 3352)	2875 (1365, 4700)	0.003*
24-h urine urea/creatinine	31.2 ± 10.1	33.0 ± 11.8	30.2 ± 6.7	33.7 ± 12.2	29.6 ± 11.3	27.6 ± 8.1	0.070
24-h urine volume, L	1.7 (1.3, 2.2)	1.7 (1.3, 2.3)	1.8 (1.2, 2.0)	1.8 (1.5, 2.4)	1.7 (1.3, 2.4)	1.6 (1.0, 2.0)	0.320
Medication history							
NSAIDs	23 (0.12)	3 (0.06)	7 (0.21)	5 (0.14)	4 (0.11)	4 (0.11)	0.403
Diuretic	78 (0.42)	18 (0.4)	14 (0.42)	13 (0.36)	18 (0.53)	15 (0.39)	0.766
RASi	88 (0.47)	31 (0.69)	22 (0.67)	20 (0.56)	11 (0.32)	4 (0.11)	<0.001*
OHD	47 (0.25)	8 (0.18)	4 (0.12)	14 (0.39)	9 (0.26)	12 (0.32)	0.039
Insulin	48 (0.26)	4 (0.09)	3 (0.09)	11 (0.31)	14 (0.41)	16 (0.42)	<0.001*

BMI: body mass index; eGFR-EPI: estimated glomerular filtration rate calculated by EPI equation; NSAIDs: non-steroidal anti-inflammatory drug; RASi: Renin-Angiotensin System inhibitor; OHD: oral hypoglycemic drugs.

*p < 0.05.

### Kidney clearances, serum and urine concentrations of PBUTs

**Figure 2.**
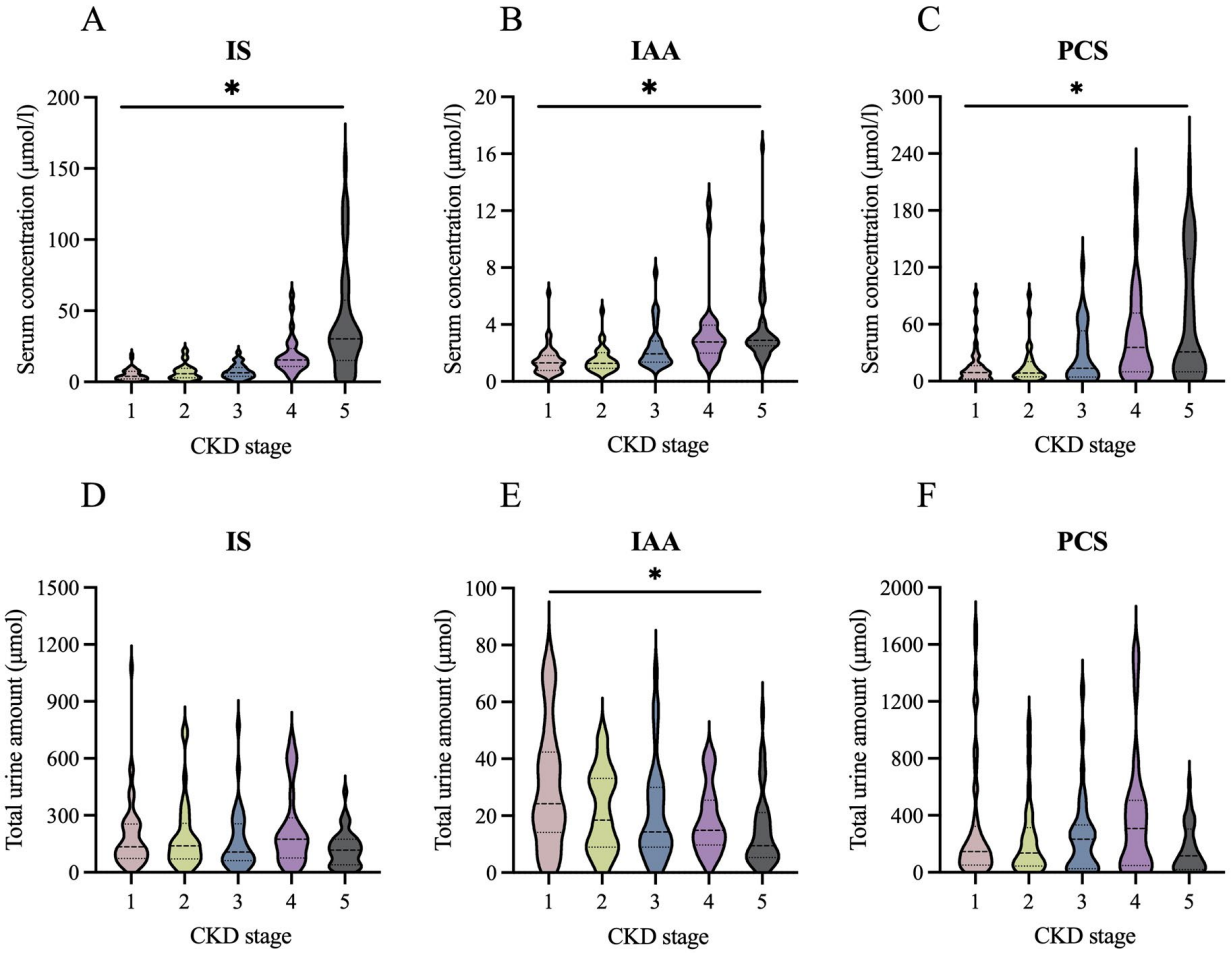
Concentrations of total IS, IAA, and pCS in serum (A–C) and urine (D–F) in CKD stages. *p < 0.05.

Serum concentrations of quantified PBUTs demonstrated a progressive increase from CKD1 to CKD5 (Figure 2 and Supplementary Table 1), as illustrated in Figure 2 and Supplementary Table 1. In contrast, analysis of 24 h urine samples revealed no significant differences in the total excretions or the concentrations of IS and pCS among CKD stages. Notably, urinary IAA was highest in CKD1 and lowest in CKD5.

**Figure 3.**
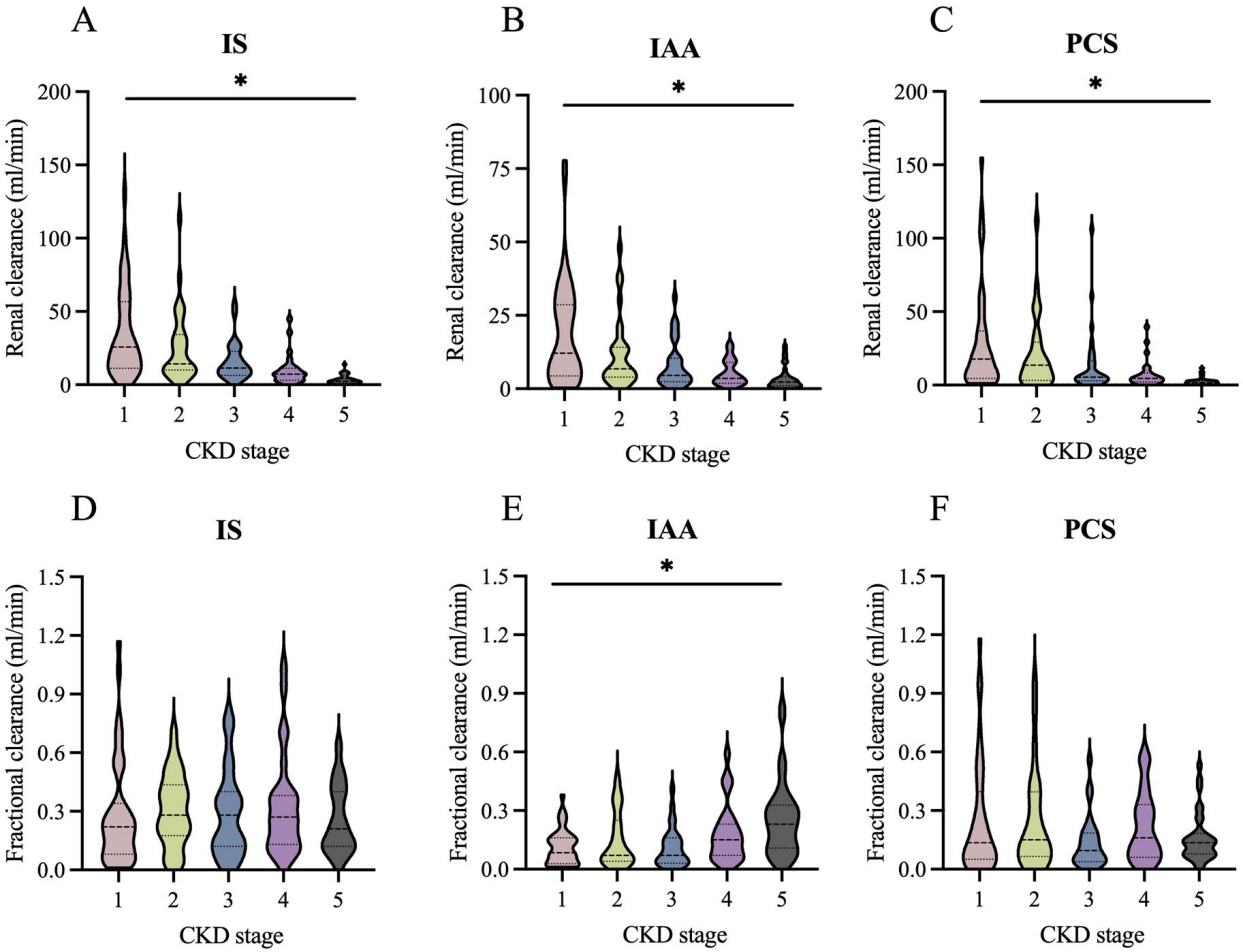
Kidney clearances of IS, IAA, and pCS in CKD stages. (A) 24 h kidney clearance of IS; (B) 24 h kidney clearance of IAA; (C) 24 h kidney clearance of pCS; (D) fractional clearance of IS; (E) fractional clearance of IAA; (F) fractional clearance of pCS; *p < 0.05.

The 24 h kidney clearance of IS (Cis) was significantly >24 h kidney clearances of IAA (Ciaa) and pCS (Cpcs) in patients with CKD1 to CKD4, as depicted in Figure 3 and Supplementary Table 1. However, in CKD stage 5, the clearances of IS, IAA, and pCS were comparable, each ∼2.0 mL/min. Substantial interindividual variation in Cis, Ciaa, and Cpcs was observed in the study, especially among patients with CKD1 to CKD3. As CKD advanced, there was a gradual decline in Cis, Ciaa, and Cpcs levels. In patients with stage 5 CKD (CKD5), the levels of Cis, Ciaa, and Cpcs were reduced to 10% to 17% of those observed in stage 1 CKD (CKD1).

Cis, Ciaa, and Cpcs demonstrated modestly intercorrelations (Cis-Cpcs, *r* = 0.3; Ciaa-Cpcs, *r* = 0.3; Cis-Ciaa, *r* = 0.6; *p*-values for all correlations <0.001). Furthermore, the correlations of Cis and Ciaa with eGFR were more pronounced than that of Cpcs with eGFR (Cis-eGFR, *r* = 0.5; Ciaa-eGFR, *r* = 0.5; Cpcs-eGFR, *r* = 0.3), with all correlations being statistically significant (*p* < 0.001).

In addition, fractional clearance of IAA (FCiaa) was statistically elevated in patients with CKD5 compared to those with CKD1-3, respectively (*p* < 0.05). However, there was no difference in fractional clearance of IS (FCis) or pCS (FCpcs) across the various CKD stages. Additionally, no linear correlation was identified between FCis, FCiaa, or FCpcs and the decline in kidney filtration rate, as measured by eGFR.

### PBUTs in different urinary protein excretion

**Figure 4.**
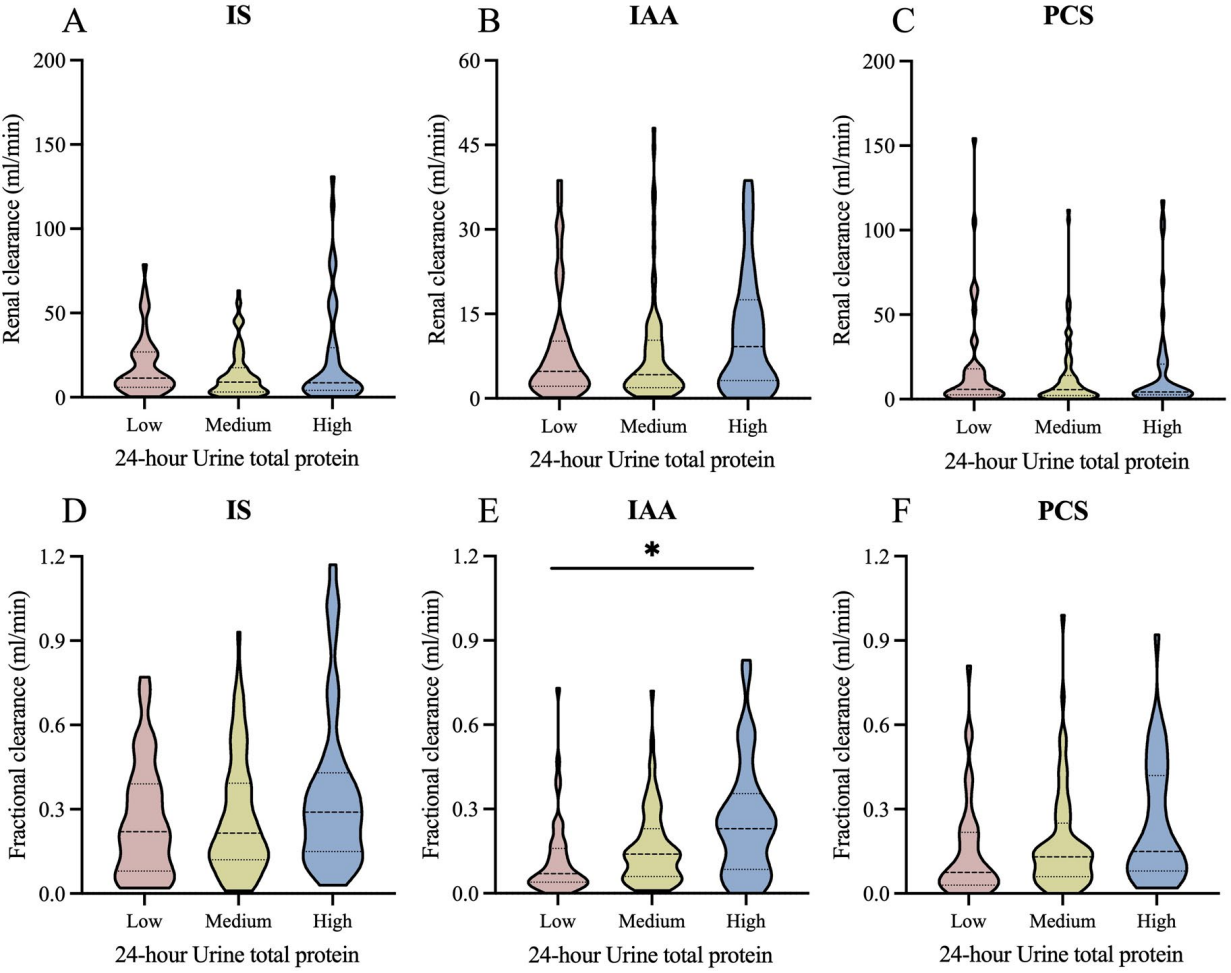
Kidney clearances and fractional clearances of PBUTs in different urinary protein excretion; *p < 0.05.

The patients were categorized into three groups based on the 24 h urinary protein excretion (UP): low-UP group (24 h UP < 500 mg), medium-UP group (500 mg ≤ 24 h UP < 3500 mg), and high-UP group (24 h UP ≥ 3500 mg). The subgroup analysis was performed to observe kidney clearances and fractional clearances of IS, IAA, and pCS. There were no significant variations in Cpbuts or FCpbuts across different protein excretion subgroups (Figure 4), with the exception of FCiaa, which exhibited an increase corresponding to higher levels of proteinuria (Supplementary Table 2).

### Association between kidney clearances of PBUTs and clinical prognosis

The median follow-up time was 58 (50, 83) months by June 2025. There were fifty-four renal events (primary end points) and 146 hospitalization events (Figure 1).

**Figure 5.**
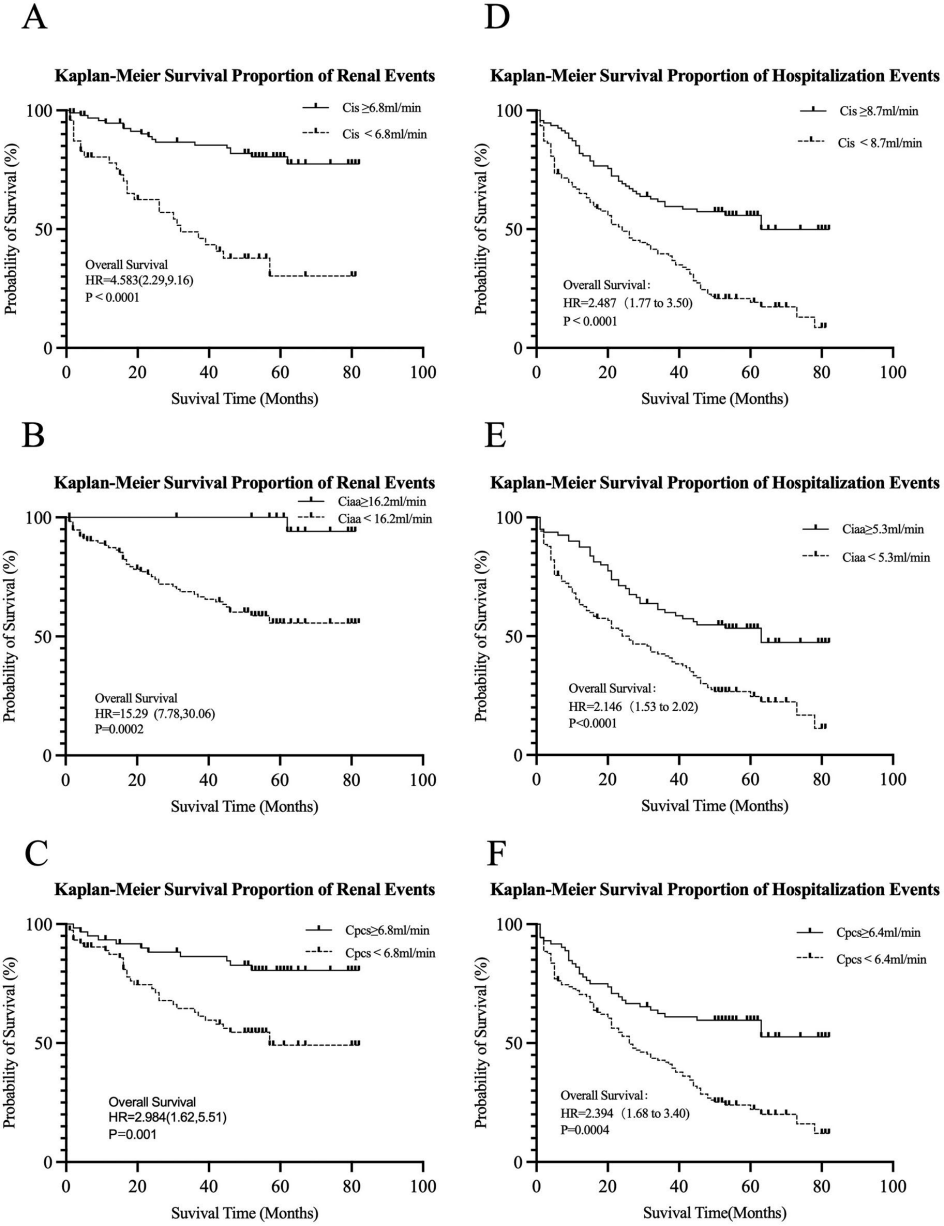
Kaplan-Meier proportion of patients with renal events (A–C) and hospitalization (D–F) according to the priori-selected groups of 24 h kidney clearances of PBUTs in patients with CKD. The cut-offs of Cis, Ciaa, and Cpcs for renal events are 6.8, 16.2, and 6.8 mL/min, respectively, and those for hospitalization events are 8.7, 5.3, and 6.4 mL/min, respectively.

According to the Kaplan-Meier curves presented in Figure 5, significant differences were observed in the incidence of renal events and hospitalization between patients with higher Cis, Ciaa, or Cpcs and those with lower levels, as classified by the optimal cutoffs determined from the ROC curve analysis.

Following univariate Cox regression analysis (Supplementary Table 3), age, sex, hypertension status, diabetes mellitus status, hemoglobin, serum creatinine, and 24 h urinary protein excretion were subsequently included in the multivariate Cox regression models as demographic, clinical, and laboratory covariates. The results showed that higher Cis and Ciaa were independently associated with significantly lower occurrence of renal adverse outcomes in full adjusted models (Table 2). While adjustment for baseline hemoglobin, serum creatinine, and urinary protein excretion attenuated the association of Cpcs with renal outcomes. In parallel, lower Cis, Ciaa were independent risk factors of hospitalization in patients with CKD after full adjustment (Table 3), respectively.

**Table 2.** Multivariate Cox regression analysis of renal events related to PBUTs.

Characteristics		Renal adverse outcomes	
		HR (95% CI)	*p*-Value
Cis, mL/min	Unadjusted	0.946 (0.917, 0.970)	<0.001*
	Model Cis-1	0.944 (0.912, 0.971)	<0.001*
	Model Cis-2	0.945 (0.916, 0.975)	<0.001*
	Model Cis-3	0.964 (0.939, 0.989)	0.005*
Ciaa, mL/min	Unadjusted	0.930 (0.891, 0.972)	0.001*
	Model Ciaa-1	0.922 (0.886, 0.963)	0.001*
	Model Ciaa-2	0.923 (0.876, 0.965)	0.001*
	Model Ciaa-3	0.941 (0.902, 0.981)	0.005*
Cpcs, mL/min	Unadjusted	0.970 (0.952, 0.993)	0.016
	Model Cpcs-1	0.971 (0.950, 0.992)	0.013*
	Model Cpcs-2	0.974 (0.954, 0.995)	0.015*
	Model Spcs-3	0.985 (0.969, 1.002)	0.089

Variables of model Cis-1 include age, sex and Cis. Variables of model Cis-2 include age, sex, diabetes, hypertension and Cis. Variables of model Cia-3 include age, sex, diabetes, hypertension, hemoglobin, serum creatinine, 24-h urinary protein excretion, and Cis.

Variables of model Ciaa-1 include age, sex and Ciaa. Variables of model Ciaa-2 include age, sex, diabetes, hypertension and Ciaa. Variables of model Ciaa-3 include age, sex, diabetes, hypertension, hemoglobin, serum creatinine, 24-h urinary protein excretion, and Ciaa.

Variables of model Cpcs-1 include age, sex and Cpcs. Variables of model Cpcs-2 include age, sex, diabetes, hypertension and Cpcs. Variables of model Cpcs-3 include age, sex, diabetes, hypertension, hemoglobin, serum creatinine, 24-h urinary protein excretion, and Cpcs.

Variables of model Spcs-1 include age, sex and Spcs. Variables of model Spcs-2 include age, sex, diabetes, hypertension and Spcs. Variables of model Spcs-3 include age, sex, diabetes, hypertension, hemoglobin, serum creatinine, 24-h urinary protein excretion, and Spcs.

* *p*-Values < 0.05.

**Table 3.** Multivariate Cox regression analysis of hospitalization events related to PBUTs.

Characteristics		Hospitalization	
		HR (95% CI)	*p*-Value
Cis, mL/min	Unadjusted	0.975 (0.962, 0.987)	<0.001*
	Model Cis-1	0.970 (0.961, 0.990)	<0.001*
	Model Cis-2	0.978 (0.965, 0.990)	0.001*
	Model Cis-3	0.984 (0.972, 0.997)	0.019*
Ciaa, mL/min	Unadjusted	0.950 (0.931, 0.970)	<0.001*
	Model Ciaa-1	0.951 (0.932, 0.980)	<0.001*
	Model Ciaa-2	0.951 (0.925, 0.977)	<0.001*
	Model Ciaa-3	0.961 (0.937, 0.985)	0.002*
Cpcs, mL/min	Unadjusted	0.980 (0.981, 0.992)	0.011*
	Model Cpcs-1	0.981 (0.982, 0.993)	0.014*
	Model Cpcs-2	0.989 (0.980, 0.998)	0.016*
	Model Spcs-3	0.993 (0.985, 1.001)	0.089

Variables of model Cis-1 include age, sex and Cis. Variables of model Cis-2 include age, sex, diabetes, hypertension and Cis. Variables of model Cia-3 include age, sex, diabetes, hypertension, hemoglobin, serum creatinine, 24-h urinary protein excretion, and Cis.

Variables of model Ciaa-1 include age, sex and Ciaa. Variables of model Ciaa-2 include age, sex, diabetes, hypertension and Ciaa. Variables of model Ciaa-3 include age, sex, diabetes, hypertension, hemoglobin, serum creatinine, 24-h urinary protein excretion, and Ciaa.

Variables of model Cpcs-1 include age, sex and Cpcs. Variables of model Cpcs-2 include age, sex, diabetes, hypertension and Cpcs. Variables of model Cpcs-3 include age, sex, diabetes, hypertension, hemoglobin, serum creatinine, 24-h urinary protein excretion, and Cpcs.

Variables of model Spcs-1 include age, sex and Spcs. Variables of model Spcs-2 include age, sex, diabetes, hypertension and Spcs. Variables of model Spcs-3 include age, sex, diabetes, hypertension, hemoglobin, serum creatinine, 24-h urinary protein excretion, and Spcs.

* *p*-Values < 0.05.

*Post-hoc* power analysis indicated that the statistical power for detecting the observed effect size exceeded 90% in both the renal and hospitalization event Cox regression models. The proportional hazards assumption was upheld, with global tests yielding non-significant results (all *p* > 0.05). Furthermore, the hazard ratio estimates for both Cis and Ciaa remained stable and statistically significant throughout the sequential adjustment from Model 1 to Model 3. In conclusion, the models demonstrated excellent performance and the results were robust.

## Discussion

In this cohort of Chinese patients with non-dialyzed CKD, the characteristics of PBUTs’ kidney clearances and their relationship with prognosis were investigated. This study confirmed a remarkable increase in serum concentrations of IS, IAA, and pCS with the progression of CKD staging. Notably, 24 h kidney clearances of PBUTs may serve as potential prognostic markers in CKD patients with the evidence that they were correlated with renal adverse outcomes and hospitalization, even fully adjusted for serum creatinine and proteinuria in this study.

Previous researches have demonstrated that the concentrations of serum PBUTs in patients with chronic kidney dysfunction are significantly increased, and reach the highest levels in patients with maintenance hemodialysis [9,18] (Supplementary Table 4). These findings align with the results of the present study, which observed a progressive increase in the serum concentrations of IS, IAA, and pCS from CKD1 to 4 stage, and those levels in CKD5 stage were ∼8 times, 2 times, and 4 times higher than those in CKD1 stage, respectively. The balance between production and excretion is an important reason for maintaining stable concentrations of PBUTs. It may remain controversial whether the increase of serum concentrations of PBUTs in CKD patients was the result of increase of PBUTs’ precursors produced by gut microbiota [26,29,30]. The diminished excretion of PBUTs resulting from renal dysfunction constitutes a significant factor contributing to the elevated serum levels of PBUTs [23]. While small and medium-sized molecules are mainly filtered through the glomerulus, PBUTs are actively transported and secreted by organic anion transporters (OATs) on the tubular epithelial cells due to their binding to albumin [31]. Unbound toxins exhibit competitive binding for the organic anion transporter (OAT) sites, demonstrating a higher affinity for these transport proteins relative to albumin, thereby facilitating enhanced cellular uptake of the toxins [32]. Therefore, in normal state, PBUTs in urine are in a free state, and the ratio of PBUTs in urine to total serum PBUTs reflects its clearance rate to some extent. Previous research has demonstrated that animals with impaired renal function exhibit a loss of tubular epithelial cells and a down-regulation of OATs expression [33], which may lead to the inability of renal tubules to secrete PBUTs. However, there is a paucity of studies examining the alterations in 24 h urinary excretion of PBUTs in CKD. The present study found no significant differences in the concentration or total excretion of urinary IS and pCS in patients with CKD stages, except for IAA, which showed a decrease corresponding to declining eGFR. These findings partially align with research findings of the Gryp T research group, who found no significant differences in the ratios of urine IS, IAA, or pCS to creatinine among groups in that study, while the urine pCG/creatinine ratio gradually increased with the progression of CKD [26]. These inconsistent findings indicated that the reduced renal excretion of PBUTs during CKD may not be adequately evaluated solely based on their concentrations or total amounts in urine. Our study presents evidence suggesting that kidney clearance of PBUTs may serve as a biomarker of organic anion transporter 1 and 3 (OAT1/3) function, which is crucial for drug elimination in clinical settings [34], thereby highlighting its broader significance for clinical application.

Over the past several decades, less attention has been devoted to methods for evaluating the function of the tubular secretion function. Recently, tubular function in CKD has been assessed by calculating the clearance fraction or kidney clearance rate of certain endogenous substances. These substances are predominantly excreted through the renal tubules into the final urine without undergoing any alterations in their molecular structure, including PBUTs [19,35]. There exist relatively precise detection methods for quantifying the concentration of these substances. Mair RD et al. identified 39 secreted solutes and compared their fractional clearances in advanced CKD patients (with an average eGFR of 7 ± 2 mL/min per 1.73 m^2^) with those in normal controls. The significant reduction in fractional clearances of IS (7.5 for CKD *vs.* 23.0 for controls) and pCS (4.1 *vs.* 8.6) suggested an impaired renal function in secreting many solutes in advanced CKD [35]. Gryp T et al. observed that the fractional clearances of IS and pCS in CKD5 stage were significantly lower than those in CKD2 stage, while the fractional clearance of IAA showed no difference in CKD stages [26]. In this study, no correlation was observed between the fractional clearances of IS, IAA, or pCS and eGFR among patients with CKD1 to CKD5. Unlike previous studies that used spot urine samples, this study employed 24 h urine samples to measure fractional clearances, which may account for the inconsistency in findings. However, 24 h urine samples and HPLC method were more accurate for the study of PBUTs pharmacokinetics in urine. Additionally, the 24 h kidney clearances of PBUTs demonstrated a significant positive correlation with eGFR in CKD patients in this study, and these clearances were also positively correlated with each other. The findings from this study are consistent with those from the Chronic Renal Insufficiency Cohort (CRIC) study conducted by Chen et al., which enrolled CKD patients with eGFR from 20 to 70 mL/min/1.73 m^2^. Their results showed that the median kidney clearances of IS and pCS were 34 mL/min and 10 mL/min, respectively, and identified a positive correlation between the clearance rates of these toxins (Pearson *r* = 0.75) [36]. It should be noted that in clinical trials investigating AST-120, SGLT2 inhibitors, or agents that modify the gut microbiome—which may impact renal tubular function or the availability of PBUTs—the concentrations of these toxins could serve as prognostic markers for assessing secretory function.

The potential impact of urinary protein on the kidney clearance of PBUTs remains largely unexplored, mainly due to the potential binding of PBUTs to albumin present in urine. To our knowledge, this study is the first to include patients with severe proteinuria. Our findings indicated that neither the clearance of indoxyl sulfate (Cis) nor p-cresyl sulfate (Cpcs) differed significantly among patients stratified by different levels of 24 h proteinuria, except that Ciaa was correlated with 24 h proteinuria in CKD1 stage and CKD5 stage. It is well known that there is a close relationship between serum albumin and the pharmacokinetics of PBUTs [37]. Studies indicated that filtered albumin is retrieved in the proximal tubule *via* three pathways: receptor-mediated endocytosis *via* cubilin (high affinity), megalin (low affinity), and fluid-phase uptake, and megalin-mediated uptake predominates under nephrotic conditions [38,39]. However, there is a paucity of relative research regarding the ability of PBUTs to bind to albumin in urine, despite the presence of intact filtered albumin in the lumen. Proteinuria also reflects the impairment of glomerular filtration [40]. This impairment may contribute to the diminished correlation observed between urinary protein excretion and kidney clearance of PBUTs following adjustments for eGFR.

The interconnections between renal and cardiovascular diseases are multifaceted, resulting in a compounding effect as both conditions advance [41]. Numerous retained PBUTs, such as IS and pCS, demonstrated to contributed to CKD progression and have been associated with cardiovascular events and increased mortality [42,43]. They are implicated with endothelial injuries, vascular calcification, leukocyte activation and adhesion, Insulin Resistance, and tissue remodeling through the induction of oxidative stress, inflammation, and fibrosis [41,44,45]. However, some studies have not found a significant association between elevated blood concentrations of PBUTs and CKD progression, all-cause mortality, or the incidence of new cardiovascular events [22] (Supplementary Table 4). On the contrary, the findings of this study demonstrated the predictive capacity of serum concentrations of IS and IAA for renal adverse outcomes. Instead, studies identified the capacity for proximal tubule secretion which plays a crucial role in the elimination of endogenous substances, including PBUTs and pharmaceuticals, as being associated with adverse outcomes in patients with CKD [18,19] (Supplementary Table 4). Notably, reduced kidney clearance of pCS or hippurate was linked to an increased risk of mortality, independent of eGFR, in a CKD cohort utilizing overnight urine samples (IQR, 11–14 h), but HR of lower IS clearance did not reach statistical significance [19]. Following adjustments for age, baseline eGFR, 24 h urinary albumin excretion, and comorbidities, reduced 24 h kidney clearance of six secretory solutes, including IS, was significantly associated with an increased risk of CKD progression. The effect sizes ranged from an 11 to 21% increased risk per 50% reduction in clearance, as observed in the CRIC study, which enrolled patients with mild-to-moderate CKD. However, the relationship between kidney clearance of secretory solutes and the incidence of heart failure or myocardial infarction was diminished after adjusting for eGFR [22]. In this study, which included patients with CKD from stage 1 to stage 5, the 24 h kidney clearances of IS and IAA were independently correlated with the incidence of renal adverse outcomes and hospitalization, even after full adjustment for serum creatinine. These findings indicate that assessing tubular secretion, a critical component of kidney function evaluation, may offer supplementary insights into CKD progression and adverse outcomes beyond the traditional measures of glomerular filtration.

This observational study is subject to several limitations. Firstly, there is presently no gold-standard method for measuring tubular secretion. The total kidney clearance rates of solutes were used instead, with the estimation assuming steady-state conditions and negligible non-kidney clearance. The unbound portion of these solutes may exert the major effect. Assessing serum concentrations of PBUTs through multiple blood samples may enhance the precision of evaluating kidney clearance of PBUTs. As with most previous studies, only total concentrations of PBUTs were measured in this study. Results may differ in some respects when the kidney clearances of PBUTs are calculated using free concentrations of PBUTs. A further limitation involves the use of the CKD-EPI equation, rather than measured GFR. Additionally, this study did not demonstrate a relationship between tubular secretory clearance and survival, potentially due to the single-center, small sample size, relatively short follow-up period, and low mortality rate. These findings should be validated in larger-scale studies.

In conclusion, the kidney clearances of IS, IAA, and pCS were rarely affected by urinary protein excretion. Lower kidney clearances of IS and IAA were independently associated with higher risks of renal adverse outcomes and hospitalization in the cohort study of CKD from stage 1 to stage 5. This study introduces a novel methodology for the risk stratification of chronic kidney disease and contributes valuable evidence to inform future precision treatment strategies.

## Supplementary Material

250830 V5 Supplementary materials.docx

## Data Availability

The datasets used and/or analyzed during the current study are available from the corresponding author on reasonable request. All data are included in the manuscript and Supplementary Material.
