# Supplementary material for: Kidney clearances of protein-bound uremic toxins predict outcomes in chronic kidney disease: a prospective cohort study
Source: Ren Fail. 2025 Nov 3;47(1):2578418. doi: 10.1080/0886022X.2025.2578418 (PMC12584829; doi:10.1080/0886022X.2025.2578418)
Supplement: 250830 V5 Supplementary materials.docx [file IRNF_A_2578418_SM8989.docx]

**Supplementary materials**

Supplementary Table 1. Serum, urine concentrations and kidney clearances of PBUTs per CKD stage

Supplementary Table 2. Clearances and fractional clearances of PBUTs in groups of 24-hour urinary protein excretion

Supplementary Table 3. Univariate Cox regression analysis of renal events and hospitalization events.

Supplementary Table 4. Summary of cohort studies related to prognosis.

Supplementary Figure1. Receiver operating characteristic (ROC) curves of cPBUTs and Kaplan-Meier proportion of surviving patients of hospitalization events in groups of different cPBUTs.

**Supplementary Table 1. Serum, urine concentrations and kidney clearances of PBUTs per CKD stage**

| **PBUTs** | **Total**  **n=186** | **CKD1**  **n=45** | **CKD2**  **n=33** | **CKD3**  **n=36** | **CKD4**  **n=34** | **CKD5**  **n=38** | ***P value*** |
| --- | --- | --- | --- | --- | --- | --- | --- |
| Serum, µmol/l |  |  |  |  |  |  |  |
| IS | 8.4 (3.7, 18.2) | 3.9 (2.0, 7.5) | 5.4 (2.7, 8.8) | 6.9 (4.1, 12.2) | 14.5 (9.7, 21.2) | 31.6 (23.0, 64.5) | **<0.001** |
| IAA | 2.0 (1.3, 3.1) | 1.3 (0.8, 1.9) | 1.3 (1.0, 1.9) | 2.0 (1.4, 2.9) | 2.7 (2.0, 4.0) | 3.1 (2.5, 5.7) | **<0.001** |
| pCS | 15.6 (6.2, 53.0) | 9.0 (2.2, 16.6) | 7.7 (4.6, 21.9) | 16.4 (7.0, 42.1) | 35.6 (9.0, 75.2) | 32.7 (14.4, 142.7) | **<0.001** |
| Urine |  |  |  |  |  |  |  |
| IS, µmol/l | 70.9 (38.7, 157.7) | 70.7 (39.7, 189.1) | 88.5 (42.8, 174.4) | 63.4 (35.7, 131.9) | 76.1 (36.2, 204.1) | 84.5 (36.7, 136.8) | 0.794 |
| IS excretion, µmol | 124.6 (67.7, 230.7) | 125.1 (72.8, 253.7) | 138.6 (73.4, 260.4) | 115.5 (65.7, 225.9) | 145.9 (52.3, 241.5) | 120.3 (65.8, 177.7) | 0.749 |
| IAA, µmol/l | 9.7 (5.2, 16.7) | 14.3 (7.6, 24.9) | 9.9 (5.9, 16.5) | 9.5 (4.4, 14.8) | 9.8 (5.2, 18.4) | 6.4 (3.4, 10.7) | **0.006** |
| IAA excretion, µmol | 15.8 (8.3, 30,7) | 23.1 (14.2, 40.9) | 17.8 (7.9, 32.8) | 14.3 (9.0, 31.6) | 14.8 (9.3, 31.5) | 9.4 (4.7, 19.3) | **0.004** |
| pCS, µmol/l | 95.4 (23.6, 204.7) | 86.1 (28.1, 239.4) | 70.2 (26.7, 194.0) | 107.9 (26.0, 200.0) | 107.8 (22.0, 275.1) | 99.0 (19.1, 183.8) | 0.921 |
| pCS excretion, µmol | 148.6 (42.7, 338.9) | 134.5 (46.8, 294.0) | 136.5 (39.3, 314.0) | 206.1 (41.6, 339.7) | 221.3 (30.6, 495.0) | 122.0 (32.0, 308.7) | 0.740 |
| Kidney clearances |  |  |  |  |  |  |  |
| Cis, ml/min | 10.0 (3.7, 24.1) | 26.7 (11.2, 58.1) | 15.8 (10.3, 33.5) | 11.4 (6.1, 18.6) | 7.0 (3.1, 10.5) | 2.2 (1.2, 3.6) | **<0.001** |
| Ciaa, ml/min | 4.5 (2.0, 12.4) | 12.1 (4.9, 29.7) | 6.8 (3.7, 14.0) | 4.5 (2.7, 9.9) | 3.4 (1.8, 8.9) | 2.1 (0.9, 3.7) | **<0.001** |
| Cpcs, ml/min | 5.3 (2.3, 17.8) | 18.4 (5.5, 41.0) | 12.5 (2.8, 25.2) | 5.5 (2.9, 15.7) | 4.1 (1.5, 8.1) | 1.8 (0.8, 3.0) | **<0.001** |
| FCis | 0.27 (0.13, 0.50) | 0.25 (0.12, 0.52) | 0.28 (0.15, 0.51) | 0.23 (0.14, 0.48) | 0.32 (0.17, 0.55) | 0.23 (0.12, 0.42) | 0.934 |
| FCiaa | 0.15 (0.07, 0.29) | 0.13 (0.05, 0.23) | 0.13 (0.05, 0.29) | 0.10 (0.04, 0.18) | 0.18 (0.07, 0.32) | 0.27 (0.11, 0.42) | **0.015** |
| FCpcs | 0.15 (0.08, 0.37) | 0.21 (0.05, 0.52) | 0.17 (0.07, 0.40) | 0.12 (0.08, 0.35) | 0.17 (0.09, 0.33) | 0.15 (0.10, 0.27) | 0.680 |
| Ccr | 42.1 (19.5, 80.4) | 109.0 (73.9, 154.2) | 67.1 (54.8, 80.8) | 44.7 (32.7, 69.0) | 21.2 (16.6, 32.8) | 8.8 (5.6, 14.8) | **<0.001** |
| eGFR-EPI | 53.8±39.1 | 109.5±11.9 | 74.4±9.2 | 43.6±8.5 | 21.2±4.4 | 8.8±3.2 | **<0.001** |

Cis, IS clearance；FCis, fractional clearance of IS; Ciaa, IAA clearance；FCiaa, fractional clearance of IAA; Cpcs, pCS clearance；FCpcs, fractional clearance of pCS; **P* <0.05

**Supplementary Table 2. Clearances and fractional clearances of PBUTs in groups of 24-hour urinary protein excretion**

|  | **UP < 0.5g**  n = 57 | **UP ≥0.5g & <3.5 g**  n = 88 | **UP ≥ 3.5 g**  n = 41 | ***P*** |
| --- | --- | --- | --- | --- |
| Cis, ml/min | 11.3(5.9, 26.8) | 9.5(3.1,17.5) | 8.5(4.1,35.7) | 0.193 |
| Ciaa, ml/min | 4.8(2.1,10.1) | 4.3(1.9,10.5) | 9.2(3.1,19.3) | 0.076 |
| Cpcs, ml/min | 5.8(2.6,18.4) | 5.4(2.21,13.9) | 4.6(2.5,21.1) | 0.664 |
| FCis | 0.3(0.1,0.6) | 0.4(0.2,0.6) | 0.6(0.3,1.2) | 0.062 |
| FCiaa | 0.1(0.05,0.3) ** | 0.2(0.1,0.3) ** | 0.3(0.2,0.9) ** | <0.001^*^ |
| FCpcs | 0.2(0.08,0.5) | 0.2(0.09,0.5) | 0.4(0.2,0.9) | 0.264 |

Cis, IS clearance；FCis, fractional clearance of IS; Ciaa, IAA clearance；FCiaa, fractional clearance of IAA; Cpcs, pCS clearance；FCpcs, fractional clearance of pCS; **, *P* <0.05 after the Kruskal-Wallis test ;*, post-hoc pairwise comparisons with Bonferroni correction, adjusted p < 0.05.

Supplementary Table 3. Univariate Cox regression analysis of renal events and hospitalization events.

| Characteristics | Renal Events | | Hospitalization Events | |
| --- | --- | --- | --- | --- |
|  | HR (95% CI) | P Value | HR (95% CI) | P Value |
| Female | 0.63(0.35,1.15) | 0.134 | 0.62(0.44,0.89) | 0.011 |
| Age, yr | 1.02(0.99,1.03) | 0.067 | 1.03(1.01,1.04) | ＜0.001 |
| DM | 1.99(1.16,3.4) | 0.011 | 2.17(1.55,3.05) | ＜0.001 |
| BMI, kg/m^2^ | 0.97(0.9,1.04) | 0.379 | 0.97(0.93,1.02) | 0.357 |
| Hypertesion | 2.95(1.44,6.07) | 0.003 | 3.42(2.03,5.77) | ＜0.001 |
| Smoking | 0.93(0.37,2.33) | 0.873 | 0.88(0.49,1.59) | 0.668 |
| Hemoglobin, g/L | 0.97(0.96,0.98) | ＜0.001 | 0.98(0.97,0.99) | ＜0.001 |
| Albumin, g/L | 1.01(0.97,1.05) | 0.625 | 0.99(0.97,1.02) | 0.772 |
| 24-hour urinary protein, g | 1.08(1.02,1.14) | 0.008 | 1.05(1.00,1.09) | 0.023 |
| Serum creatine, μmol/L | 1.004(1.003,1.005) | ＜0.001 | 1.002(1.001,1.003) | ＜0.001 |
| 24-hour urine Urea/Creatinine | 0.977(0.947,1.008) | 0.139 | 0.994(0.97,1.02) | 0.637 |
| Clearances of PBUTs |  |  |  |  |
| Cis, ml/min | 0.946(0.917,0.97) | ＜0.001 | 0.975(0.962,0.987) | ＜0.001 |
| FCis | 0.96(0.59,1.55) | 0.872 | 0.98(0.72,1.34) | 0.933 |
| Ciaa, ml/min | 0.93(0.89,0.97) | 0.001 | 0.95(0.93,0.97) | ＜0.001 |
| FCiaa | 1.57(0.86,2.85) | 0.135 | 1.26(0.88,1.82) | 0.206 |
| Cpcs, ml/min | 0.97(0.95,0.99) | 0.016 | 0.98(0.98,0.99) | 0.011 |
| FCpcs | 1.1(0.95,1.35) | 0.17 | 0.91(0.76,1.08) | 0.300 |

BMI, body mass index; DM, diabetes mellitus; eGFR, estimated glomerular filtration rate.

Supplementary Table 4. Summary of cohort studies related to prognosis.

| Authors | Sizes | HR (95% CI) | Event rates | Methods to calculate the PBUTs clearance | Patient Group |
| --- | --- | --- | --- | --- | --- |
| Ruben Poesen et al^1^ | 488 | Mortality: Higher serum p–cresol, 1.58(1.10 to 2.29);  lower proportion of serum p–cresyl sulfate to glucuronide:0.65 (0.47 to 0.89).  cardiovascular disease: Higher serum p–cresol, 1.68 (1.27 to 2.22); lower proportion of serum p–cresyl sulfate to glucuronide: 0.55 (0.42 to 0.72). | N/A | N/A | CKD stages 1–5. |
| Yan Chen et al^2^ | 3,407 | Secretory solute clearances and incident myocardial for indoxyl sulfate: 0.85 (0.73-1.00, p=0.05). | 310 (over a median follow-up of 9.2 years). | Standardized clearance = ln(secretory clearance) – min(ln(secretory clearance)) * 100  range(ln(secretory clearance))  where ln(clearance) is the kidney clearance of each secretory solutes after log-transformation,  min(ln(clearance)) is the minimum value in the distribution, and range(ln(clearance)) is the  difference between the maximum and minimum values in the distribution. | The Chronic Renal Insufficiency Cohort (CRIC) study.  a mean age of 56 years; 45% women; 41% Black;  and a median eGFR of 43 mL/min/1.73m^2^. |
| Astrid M. Suchy-Dicey et al^3^ | 298 | Low clearance of hippurate or p-cresol sulfate  associated with greater risk of death independent of eGFR: hazard ratio, 2.3 (1.1-4.7); hazard ratio, 2.5 (1.0 to 6.1). | 43 During a median of 3.0 years of follow-up (IQR, 1.8–4.0 years). | N/A | The median eGFR was 41.8 ml/min per 1.73 m2 (interquartile range [IQR], 28.5–59.9 ml/min per 1.73 m^2^). |

N/A, not application.

**References:**

1. Poesen R, Evenepoel P, de Loor H, Kuypers D, Augustijns P, Meijers B. Metabolism, Protein Binding, and Renal Clearance of Microbiota-Derived p-Cresol in Patients with CKD. Clin J Am Soc Nephrol 2016;11:1136-1144.

2. Chen Y, Zelnick LR, Huber MP, Wang K, Bansal N, Hoofnagle AN, Paranji RK, Heckbert SR, Weiss NS, Go AS, Hsu CY, Feldman HI, Waikar SS, Mehta RC, Srivastava A, Seliger SL, Lash JP, Porter AC, Raj DS, Kestenbaum BR. Association Between Kidney Clearance of Secretory Solutes and Cardiovascular Events: The Chronic Renal Insufficiency Cohort (CRIC) Study. Am J Kidney Dis 2021;78:226-235.e1.

3. Suchy-Dicey AM, Laha T, Hoofnagle A, Newitt R, Sirich TL, Meyer TW, Thummel KE, Yanez ND, Himmelfarb J, Weiss NS, Kestenbaum BR. Tubular Secretion in CKD. J Am Soc Nephrol 2016;27:2148-55.


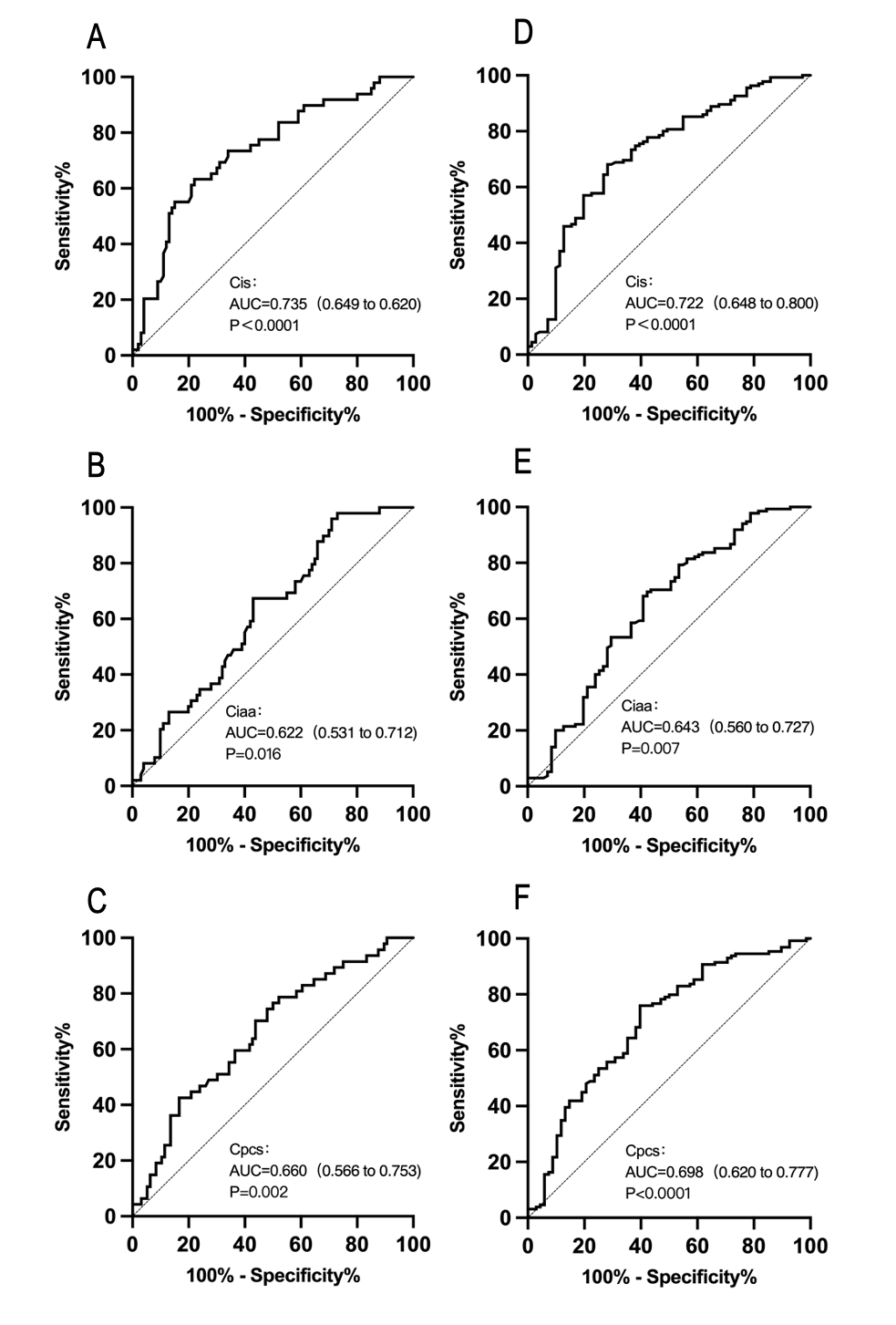


Supplementary Figure1. Receiver operating characteristic (ROC) curves of kidney clearances of PBUTs in predicting renal events (A-C) and hospitalization events (D –F).
